# Video-assisted thoracoscopic surgery versus robotic-assisted thoracoscopic surgery in the surgical treatment of Masaoka stage I thymoma

**DOI:** 10.1186/1477-7819-11-157

**Published:** 2013-07-17

**Authors:** Bo Ye, Ji-Cheng Tantai, Wang Li, Xiao-Xiao Ge, Jian Feng, Ming Cheng, Heng Zhao

**Affiliations:** 1 Department of Thoracic Surgery, Shanghai Chest Hospital, Shanghai Jiaotong University, Shanghai, Huaihaixi Road 241, 200030, P.R China; 2 Renji-MedX Clinical Stem Cell Research Center, Renji Hospital, Shanghai Jiao Tong University School of Medicine, Shanghai 200127, PR China

**Keywords:** Robotics, Thymoma, Minimally invasive surgery, Thymus

## Abstract

**Background:**

The purpose of this study was to compare perioperative outcomes in patients who underwent video-assisted thoracoscopic surgery or robot-assisted thoracoscopic surgery and assess the feasibility of robotic-assisted thymectomy for the treatment of Masaoka stage I.

**Methods:**

We evaluated the short-term outcomes of 46 patients who underwent surgery for Masaoka stage I thymoma without myasthenia gravis between January 2009 and June 2012. Of these patients, 25 received unilateral video-assisted thoracoscopic surgery (VATS group) and the rest 21 recieved unilateral robotic-assisted thoracoscopic surgery (RATS group). We evaluated the duration of surgery, amount of intraoperative blood loss, duration of chest drainage, duration of postoperative hospital stay, hospitalization costs, postoperative complications and oncological outcomes.

**Results:**

The duration of surgery was not significantly different between the two groups. Intraoperative blood loss volumes did not differ significantly between the VATS and RATS groups (86.8 mL and 58.6 mL, respectively; *P*=0.168). The postoperative hospital stay was significantly shorter in the RATS group (3.7 days *vs.* 6.7 days; *P* <0.01), and the postoperative pleural drainage volume of the RATS group was significantly less than VATS group (1.1 days *vs.* 3.6 days; *P* <0.01). No patients in the RATS group needed conversion to open surgery. However, in the VATS series, one patient had conversion to an open procedure. No surgical complications were observed except that one case had pulmonary atelectasis in the RATS group and one case developed pneumonia after surgery. Use of robot is much more expensive than video. No early recurrence was observed in both groups.

**Conclusions:**

Robotic thymectomy is feasible and safe for Masaoka stage I thymoma. RATS is equally minimally invasive as VATS and results in a shorter drainage period and reduced hospital stay compared with the VATS approach.

## Background

Thymomas are rare intrathoracic neoplasms of the thymus with an annual incidence of approximately 0.15 per 100,000 person-years [1]. Surgical intervention remains the only curative treatment and is traditionally performed through a median sternotomy, with complete resection of the tumor, thymus, thymic cervical extensions, and the surrounding perithymic fat [2,3]. With the advent of improved optics and computer-assisted surgical systems, minimally invasive thymectomies by video-assisted or robotic techniques are becoming increasingly popular [4].

We herein present our operative method of unilateral RATS thymectomy for the treatment of Masaoka stage I thymoma without myasthenia gravis. We compared the short-term outcomes of patients undergoing RATS and those undergoing VATS thymectomy.

## Methods

We retrospectively reviewed our experience on thymectomy for the treatment of stage I thymoma at the Shanghai Chest Hospital, Shanghai Jiaotong University. Between January 2009 and June 2012, a total of 532 patients underwent resection of primary thymic tumors with curative intent. Of these patients, 46 (22 men and 24 women) with a pathological diagnosis of Masaoka stage I thymoma without myasthenia gravis who consecutively underwent VATS or RATS were selected for this study. The performance of VATS and RATS was approved by hospital local ethics committee, and written informed consents were obtained from all patients. Twenty-five patients underwent thymoma resection using VATS, and the other 21 underwent RATS.

In each case, preoperative computed tomography (CT) demonstrated that the anterior mediastinal tumor, which was histologically confirmed as thymoma after surgery, demonstrated features highly suggestive of thymoma without signs of invasion to the surrounding structures. VATS or RATS was indicated for thymomas of <5 cm.

All patients were anesthetized using a double-lumen tube for split-lung ventilation and were placed left- or right-side up at 30°. For VATS, the diseased side of the thoracic cavity was approached using a three-port technique. The diseased side of the thymus was dissected from the inferior pole to the superior pole, and the contralateral thymus was dissected after the left brachiocephalic vein had been exposed. The thymic veins were divided with ultrasonic shears or electric cautery for dissection. The procedure in the VATS group was conventional.

For RATS, the first incision was generally performed in the fifth intercostal space at the anterior axillary line. Thus, the camera was inserted to explore the chest cavity, and carbon dioxide was inflated (ranging between 4 and 8_mmHg) to enlarge the operating space and safely perform the other port incisions: at the anterior axillary line at the third intercostal and at the fifth intercostal space at the mid-clavicular line. The operator’s right hand held ultrasonic shears to perform the dissection, and the left hand held Cadiere forceps (EndoWrist; Intuitive Surgical, Inc, Sunnyvale, CA, USA), an atraumatic instrument for grasping the normal thymus. The normal thymic tissue and perithymic fat were used to grasp and place traction on the tumor, avoiding direct manipulation of the tumor to minimize the risk of capsule damage.

After inspection of the thymus gland and the thymoma, the dissection generally started inferiorly, first from the left side at the pericardiophrenic angle; it then continued on the right side, from the retrosternal area. The right mediastinal pleura and right phrenic nerve were identified, permitting safe dissection of the right inferior horn under direct visualization of the nerve. Consequently, the dissection continued upward to the neck until the superior horns were identified. The thymic veins were identified and separately clipped. The lesions were removed with endoscopic pouches from the cavity through the port incision in the mid-axillary region. If necessary, the incision was enlarged (3 cm maximum). No additional utility incision was used. A drainage tube was inserted, generally 32 inches. All thymus tissue and perithymic fat were dissected according to the Masaoka criteria [5], and the completeness of the thymectomy was assessed by macroscopic inspection of the thymic bed and specimen.

We evaluated the duration of surgery, amount of intraoperative blood loss, duration of chest drainage, duration of postoperative hospital stay, postoperative complications, and hospitalization costs. The diagnoses of all resected thymomas, surgical margins, and Masaoka stage were confirmed by histological examination. Size, as shown by the maximal diameter of the tumor within the resected specimen, was measured. Recurrence of thymoma was evaluated by CT of the chest 6 and 12months after surgery and once yearly thereafter.

### Statistical analysis

The data are reported as means±standard deviations. Statistical analysis was performed with SPSS 16.0 statistical software (SPSS Inc., Chicago, IL, USA). Probability values were generated with the chi-squared test (categorical data) and the unpaired *t*-test (noncategorical data). A probability value of *P* <0.05 was considered to be statistically significant.

## Results

The preoperative characteristics of the two groups of patients are shown in Table 1. The VATS group comprised 13 men and 12 women with a mean age of 53.3 years. The RATS group included 9 men and 12 women with a mean age of 52.7 years. In the VATS group, 16 patients were approached from the right thoracic cavity, and nine patients were approached from the left. Surgical outcomes in the VATS and RATS groups are shown in Table 2. The duration of surgery and volume of intraoperative blood loss were not significantly different between the two groups. The mean postoperative hospital stay was shorter in the RATS group (3.7 days in the RATS group compared with 6.7 days in the VATS group; *P* <0.01). The median postoperative stay was shorter in the RATS group (4 days *vs.* 7 days, *P* <0.01). The criteria for chest tube removal were similar for both groups. Chest tubes were removed when the drainage output volume declined to <100 mL in a 24-h period. The mean postoperative pleural drainage volume was significantly decreased in the RATS group (1.1 days *vs.* 3.6 days; *P* <0.01). However, the mean hospitalcosts were higher in the RATS group (USD 6,097±1,342 *vs.* USD 8662±2,375 *P* <0.01) possibly because of expense of equipment.

**Table 1:** Characteristics of the patients in the VATS and RATS groups

	**VATS**	**RATS**	***P *value**
Patients (*n*)	25	21	
Gender (male:female)	13:12	9:12	0.536
Age (mean±SD) (years)	53.4±5.4	52.7±7.8	0.74
Range (years)	(43-62)	(37-66)	
Stage (Masaoka I)	25	21	
Tumor size (mm)	30.4±7.9	29.1±7.7	0.56
Approach (right/left)	16/9	19/2	

*RATS*, robotic-assisted thoracoscopic surgery; *VATS*, video-assisted thoracoscopic surgery.

**Table 2:** Surgical outcomes of the patients in the VATS and RATS groups

	**VATS**	**RATS**	***P *value**
Patients (*n*)	25	21	
Duration of surgery (min)	103.6 ± 36	96.2 ± 39.8	0.51
Blood loss (mL)	86.8 ± 97.1	58.6 ± 20.6	0.168
Postoperative pleural drainage (days)	3.6 ± 1.2	1.1 ± 1	<0.01
Postoperative hospital stay (days)	6.7 ± 1.4	3.7 ± 1.1	<0.01
Hospitalization costs (CNY) or USD	37,376.6 ± 8,226.3 or	53,099.3 ± 14,556	<0.01
	6,097 ± 1,342	8,662 ± 2,375	
Converted to open surgery	1	0	
Blood transfusion	1	0	
Postoperative complications	1	1	

*CNY*, Chinese Yuan; *RATS*, robotic-assisted thoracoscopic surgery; *VATS*, video-assisted thoracoscopic surgery.

The da Vinci system enables surgeons to accurately dissect the left thymic lobe from right-sided access in most patients. No patients in the RATS group underwent conversion to open surgery. No surgical complications, such as massive bleeding, were observed in this series with the exception of one case of pulmonary atelectasis in a male patient 2 days after surgery.

Alternatively, in the VATS group, conversion to an open procedure was required in only one patient because of intraoperative injury of the left innominate vein and one case of postoperative pneumonia occurred. In both groups, the surgical margins were free of tumor tissue. No early recurrence was observed during the postoperative follow-up period of 17.5 months (range, 6-48 months) in the RATS group and 25.2 months (range, 6-48 months) in the VATS group.

## Discussion

The open surgical approach has generally been accepted as the gold standard for thymoma resection [6-9]. However, over the last decades, both thoracoscopic and, more recently, robotic approaches have been introduced into the thoracic field and also applied to thymectomy. Several data were published regarding minimally invasive thymectomy, reporting interesting outcomes and emphasizing less operative trauma, shorter hospital stays, preserved pulmonary function, and superior cosmetic results [10-13]. However, only a few major series have reported the surgical results of RATS surgery for thymoma [14-16]. In the surgical treatment of non-invasive thymoma, the choice of RATS or VATS remains controversial.

Our short-term surgical outcomes showed the safety and efficacy of RATS for Masaoka stage I thymoma. The duration of surgery was the same in both groups, and there was no statistically significant difference between the two groups (*P*=0.168) in the amount of blood lost. However, RATS thymectomy was less invasive, as indicated by the significant decrease in the mean or median postoperative stay in the RATS group. Furthermore, there were no serious postoperative complications in the RATS group. In addition, no case was converted to open surgery, and no case required blood transfusion. Although the follow-up period was short, there was no early recurrence of thymoma. Our results suggest that RATS thymectomy is tolerated in patients with early-stage thymoma.

An important point is that we use ultrasonic devices in RATS. Great care must be taken to avoid vascular and nervous injuries. Bleeding is the most serious complication and should be kept in mind during this operation. Special attention must be paid because venous drainage of the gland to the innominate vein involves thin vessels; each must be isolated from the thymic veins, coagulated, and cut with an ultrasonic device to minimize the amount of bleeding. In the RATS series, no patients experienced conversion to an open procedure. However, in the VATS series, one patient experienced conversion to an open procedure because of injury to the left innominate vein during electric cautery for dissection. Although small, this tool is excellent in terms of handling and indispensable for this operating method.

Nevertheless, with regard to oncological outcomes, some criticisms of minimally invasive approaches still exist. First, the possibility of tumor spread into the chest cavity has been reported in some papers [6,17,18]. Agasthian et al. [19] reported a local recurrence rate of 3.4% in patients who underwent VATS thymectomy for thymoma, while two recent papers compared VATS and open approaches for thymomas. Cheng et al. [20] observed no local or pleural disease recurrence for stage II thymoma in either the open or VATS groups. Similar results were published by Pennathur et al. [19] in a larger comparative study, reporting no significant difference in disease recurrence or overall survival between the two groups. In our small series, despite the fact that the follow-up period was short, we observed no local or pleural recurrence. We believe that the robotic system facilitates differentiation of thymoma from normal thymic tissue and allows for safe manipulation. Nevertheless, an important factor that likely affected the success of the minimally invasive approach is the dimension of the lesion. In our series, the mean thymoma dimension was 30.4±7.9 mm in VATS and 29.05±7.7 mm in RATS, similar to the dimensions reported in the above-mentioned comparative studies of the VATS approach [19,20]. However, the indolent nature of thymomatous disease necessitates a long follow-up [21] of 10 years to evaluate the survival and disease-free rates. Thus, further multi-institutional studies involving larger series are necessary.

Because high costs are one of the main points of criticism voiced in connection with robotic-assisted operations, we recently evaluated the hospitalization costs of thymectomy for various minimally invasive approaches. Use of robots is significantly more expensive, and we demonstrated high costs compared with VATS (*P* <0.01). These high costs are primarily caused by the expensive robotic instruments, which can be reused only a limited number of times [22].

The present study has some limitations, including its retrospective nature, which may have resulted in a selection bias. Moreover, the study sample was small and the follow-up period short. However, this is an initial experience resulting from gained skill in robotic surgery for thymoma, and the main aim was to analyze the safety and technical feasibility of the robotic approach for early-stage thymomas. In fact, few data regarding the robotic approach with a focus on thymoma have been published, and our small series is the largest one to our knowledge.

## Conclusion

This preliminary study demonstrates the safety and feasibility of robotic thymectomy for thymoma, with no mortality, low morbidity, and no nerve or vessel injury. RATS is equally minimally invasive as VATS and results in a shorter drainage period and reduced hospital stay compared with VATS approach. Nevertheless, it is hoped that randomized multi-institutional trials with long-term follow-up will be designed to compare the trans-sternal, video-assisted thoracoscopic, and robotic approaches and evaluate the oncological outcomes.

## Abbreviations

RATS: robotic-assisted thoracoscopic surgery; VATS: video-assisted thoracoscopic surgery.

## Competing interests

The authors declared that they have no competing interets.

## Authors’ contributions

BY and J-CT contributed equally to this work. BY, J-CT, and HZ carried out the design of the study and performed the statistical analysis. WL, X-XG, MC, and JF conceived of the study, and participated in its design and coordination and helped to draft the manuscript. All authors read and approved the final manuscript.
